# Dextran-Coated Zinc-Doped Hydroxyapatite for Biomedical Applications

**DOI:** 10.3390/polym11050886

**Published:** 2019-05-15

**Authors:** Daniela Predoi, Simona Liliana Iconaru, Mihai Valentin Predoi

**Affiliations:** 1 National Institute of Materials Physics, Atomistilor Street, No. 405A, P.O. Box MG 07, 077125 Magurele, Romania; simonaiconaru@gmail.com; 2 University Politehnica of Bucharest, BN 002, 313 Splaiul Independentei, Sector 6, 060042 Bucharest, Romania; predoi@gmail.com

**Keywords:** hydroxyapatite, zinc, dextran, sol-gel, antimicrobial properties

## Abstract

Dextran-coated zinc-doped hydroxyapatite (ZnHApD) was synthesized by an adapted sol-gel method. The stability of ZnHApD nanoparticles in an aqueous solution was analyzed using ultrasonic measurements. The analysis of the evolution in time of the attenuation for each of the frequencies was performed. The X-ray diffraction (XRD) investigations exhibited that no impurity was found. The morphology, size and size distribution of the ZnHApD sample was investigated by transmission electron microscopy (TEM) and scanning electron microscopy (SEM). The TEM and SEM results showed that the ZnHApD particles have an ellipsoidal shape and a narrow distribution of sizes. The cell growth and toxicity of HEK-293 cells were investigated on the ZnHApD solution for four different concentrations and analyzed after 24 and 48 h. The ZnHApD solution presented a non-toxic activity against HEK-293 cells for all analyzed concentrations. The antibacterial assay revealed that all the tested microorganisms were inhibited by the ZnHApD dispersion after 24 and 48 h of incubation. It was observed that the effect of the ZnHApD solution on bacteria growth depended on the bacterial strain. The *Porphyromonas gingivalis* ATCC 33277 bacterial strain was the most sensitive, as a growth inhibition in the presence of 0.075 μg/mL ZnHApD in the culture medium was observed.

## 1. Introduction

Currently one of the most pressing public health problems worldwide is the emergence of drug resistant microorganisms. Microorganism contamination is of great concern in numerous fields, such as medical devices, water purification systems, hospitals, dental office equipment, healthcare products, food packaging, food storage and household items [1,2]. At a global level, tremendous efforts are being directed towards developing new strategies for controlling bacterial activity. Several nanotechnology strategies that can inhibit cellular proliferation, adhesion and attachment based on nanomaterials have been proposed [1,2,3,4,5]. Novel antimicrobial agents able to interfere in the bacterial physiology and quorum sensing, thus avoiding biofilm development, are the goal of researchers worldwide. The existing literature reported and demonstrated that metallic compounds possess antimicrobial activity and that they could help induce antimicrobial properties in various compounds [4,5,6]. Recent studies have emphasized that some nanostructured surfaces can successfully neutralize electrostatic forces to reduce cellular adhesion and inhibit biofilm formation. More than three quarters of all microbial infections are usually associated with the development of microbial biofilm [6,7,8,9], for example, oral diseases, periodontal diseases and dental caries, which are caused by the oral biofilm known as dental plaque. A report from the World Health Organization in 2005 showed that in the majority of developed countries, dental caries represents a serious health problem and affects people of all ages [10]. Studies have emphasized that due to a cariogenic diet, dental caries and affections such as periodontal disease have a significant effect on quality of life and could lead to a great deterioration of the systemic health of the population [10,11,12]. Previously, numerous conventional strategies such as anti-dental plaque strategy, protection of dental hard tissues [13], established plaque removal [14], interference with the attachment of oral microorganisms to the pellicle [15] or inhibition of growth or killing of oral microorganisms [15,16] have been developed for the treatment of oral biofilm-related diseases. Usually, oral permucosal implants pose a very high risk of developing biofilm-related infections. Studies have reported that approximately 90% of all dental implants show signs of inflammation and that from these, more than 50% show signs of tissue destruction [17,18]. The development of a multi-layered biofilm starts with the attachment of a single bacterial cell, followed by its adhesion and proliferation. Generally adhered cells suffer a modification of their gene expression until they reach a more pathogenic and resilient type. Compared with their planktonic form, sessile phenotypes have an amazing resistance to conventional antimicrobial therapies, such as antibiotics and disinfectants [18,19]. Considering these facts, an effective strategy in combating the apparition of dental implant-related infections would lead to the prevention of biofilm formation. There have previously been numerous attempts in developing therapies for the treatment of oral biofilm related diseases [13,14,15,16]. More recently, however, there has been tremendous attention given to the development of more preventive and minimally invasive strategies in the treatment of caries based on an anticariogenic diet, proper oral hygiene and the use of antimicrobial peptides, probiotics or natural substances such as plant-based extract [15,20,21]. Recent reports have emphasized that the use of enzymes has also been considered as an alternative strategy against biofilms associated with medical devices or industrial biofilms [22]. Moreover, studies have highlighted that even though the role of the enzymes in oral bacteria is not entirely understood yet, it is known that they are among the virulence factors of streptococci by facilitating the use of the dextran found in the oral cavity as an additional source of carbon for microorganisms [23,24]. In their studies, Wood and Critchley [25], Carlsson and Egelberg [26] and Gibbons and Banghart [27] concluded that the production of extracellular dextran from sucrose by cariogenic bacteria has an important role in the development of dental plaque. Moreover, dextran has been reported to possess a natural resistance to salivary microorganisms, and also that plaque bacteria could have dextranase activity [28,29]. On the other hand, Gibbons and Fitzgerald [28] described how plaque formation starts with the adherence of cariogenic streptococci onto the dextran adsorbed on the teeth’s surface. The reason why polysaccharides have the ability to adhere to the tooth surface is not yet elucidated, but there are assumptions that this is due to an existing affinity between the macromolecules of enamel and bacterial polysaccharides [25,26,27,28,29]. Dextranases are dextran-degrading enzymes, belonging to a special group of carbohydrases and transferases, and are commonly produced by oral *Streptococcus* bacteria [24,30,31]. In the past, several studies have tried to emphasize the role of dextranases in anti-caries applications, and the in vitro studies conducted, reported that these enzymes could partially hydrolyze dextrans, and also have the ability to inhibit mutan synthesis and participate in the degradation of artificially-induced mutans streptococci biofilms [32,33,34]. In the study conducted by Guggenheim et al. [35], it was concluded that fungal dextranase inhibited the development of dental caries in rats, while studies performed by Caldwell et al. [36] reported that dextranase used in the form of mouth-rinse had no visible effect on the development of plaque and plaque dry weight. In a similar study performed by Lobene [37], it was shown that dextranase helped to reduce plaque dry weight. Numerous studies have proven that enzymes have the ability to destroy the physical integrity of a bacterial biofilm matrix, making it easier to combat biofilm-related infection [9]. On the other hand, another approach in the case of the apparition of dental plaque was the use of antimicrobial agents [38]. Presently, the delivery of antiplaque agents from a dentifrice is a commonly accepted practice in order to achieve correct dental hygiene [39]. Therefore, the use of a common composite based on biomaterials, such as hydroxyapatite doped with metal ions (HAp) with antimicrobial properties, are often employed as new therapies for treating resistant microorganism-related infections. Studies have revealed that one of the best oral cavity antimicrobial agents that is tolerable for humans is zinc [40,41]. In this context, the aim of this study was the preparation of hydroxyapatite doped with zinc in a dextran matrix for biomedical applications. A material based on hydroxyapatite doped with zinc in a dextran matrix has the potential to be more useful than conventional ceramics due to the high surface area, reactivity and biomimetic morphology of the HAp nanoparticles combined with the osteoblast and bone regenerative and antimicrobial properties of zinc ions, as well as with the biological properties of dextran for biomedical applications, such as tissue engineering, fillers for composites, reparative materials, coatings for prosthetic devices and carriers for drugs. This study reports the dextran-coated zinc-doped hydroxyapatite (ZnHApD) stability in an aqueous solution using ultrasonic measurements. The powders resulting from the ZnHApD solution were analyzed using X-ray diffraction (XRD), transmission electron microscopy (TEM) and scanning electron microscopy (SEM). The adhesion and proliferation of HEK-293 cells in the presence of ZnHApD nanoparticles were investigated. The antibacterial activities of the ZnHApD samples were determined against Gram-positive and Gram-negative bacterial strains.

## 2. Materials and Methods 

### 2.1. Sample Preparation

#### 2.1.1. Materials 

To obtain zinc-doped hydroxyapatite coated with dextran solutions by the sol-gel method we used calcium nitrate (Ca(NO_3_)_2_∙4H_2_O, Sigma-Aldrich, St. Louis, MA, USA), zinc nitrate (Zn(NO_3_)_2_·6H_2_O, Alfa Aesar, Karlsruhe, Germany; 99.99% purity), ammonium hydrogen phosphate ((NH_4_)_2_HPO_4_), Alfa Aesar, Karlsruhe, Germany; 99.99% purity), triethanolamine (C_6_H_15_NO_3_, Sigma-Aldrich, St. Louis, MA, USA ≥ 99.0% (GC) purity), dextran (H(C_6_H_10_O_5_)_x_OH, (MW ∼ 40,000), Merck, Kenilworth, NJ, USA) and ethanol (C_2_H_5_OH, Merck, Kenilworth, NJ, USA). Bi-distilled water was also used in the synthesis of dextran-coated zinc-doped hydroxyapatite. 

#### 2.1.2. Dextran-Coated Zinc-Doped Hydroxyapatite 

Dextran-coated zinc-doped hydroxyapatite (ZnHApD) was prepared by an adapted sol-gel method [42]. The synthesis was carried out at a temperature of 100 °C under atmospheric pressure with a Zn/(Zn + Ca) ratio of 10%. In the first step, (NH_4_)_2_HPO_4_ was dissolved in absolute ethanol using a magnetic stirrer. In the second step, Ca(NO_3_)_2_·4H2O and Zn(NO_3_)_6_·6H_2_O were dissolved in a beaker of distilled water under continuous agitation. The first and second resulting solutions were dropped in a dextran solution (20 g in 100 mL of water) and vigorously stirred at room temperature. The molar reports Ca/P and (Ca + Zn)/P ratio was maintained at 1.67 [43,44] to keep the HAp structure. The pH value of the solution was preserved to 11, adding the NH_4_OH solution gradually. The resulting solution was stirred slowly for 6 h at 100 °C until the formation of a gel. The resulting gel was washed five times with double-distilled water and ethanol according to previous studies [45]. Finally, the resulting gel was redisposed in a dextran solution (10 g in 100 mL of water). The ZnHApD final solution was analyzed by different technics.

The chemical structure of dextran that is a neutral polysaccharide includes α- (1 → 6) linkages that may range from 97% to 50% of total glucosidic bonds [46] and according to previous studies [47], the hydroxyl groups in dextran are preferred for most derivatizations [47,48,49]. In agreement with previous studies [49], the compounds such as hydroxyapatite have high flexion due to the (Ca++) group and can interact with the (–OH) of the polymer chains. The interaction of hydroxyapatite and the linear polysaccharide is according to Humberto et al. [50].

### 2.2. Characterizations

#### 2.2.1. Ultrasonic Measurements

**Figure 1:**
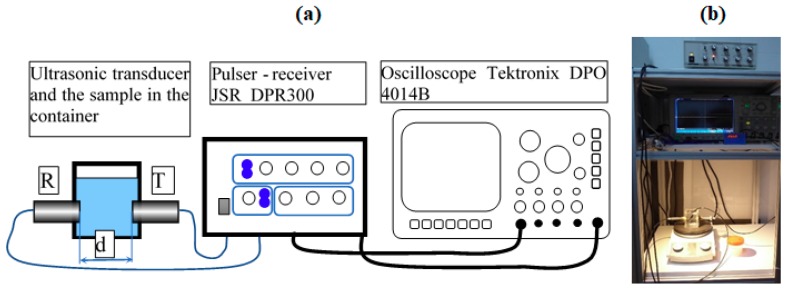
Experimental setup: schematic (**a**) and photo (**b**).

To carry out the study on the stability of ZnHApD dispersions using ultrasonic measurements, the sample was stirred at room temperature for 30 min. The system shown in Figure 1 was used to perform ultrasound studies. There are two coaxial ultrasonic transducers distanced by d = 30 mm of 5 MHz central frequency, model H5K (General-Electric, Krautkramer, Hürth, Germany). The transducer marked with R sends and receives the echoes while the T-marked transducer receives the echoes. These are general purpose longitudinal non-focusing transducers that attenuate the received echoes by different amounts at different frequencies. To minimize the errors due to losses in transducers, in the present study we investigated only the echoes received by the T-transducer and the samples were tested every 5 s.

#### 2.2.2. Structural and Morphological Characterizations. 

In order to determine the crystal structures of the ZnHApD, the XRD patterns were recorded on a Bruker D8 Advance diffractometer (Bruker, Karlsruhe, Germany) using Cu_Kα_ (λ = 1.5418 Å) radiation in the 2θ ranging from 15° to 70°. TEM micrographs were taken by a transmission electron microscope (JEM 2100, JEOL, Akishima, Tokyo, Japan). To analyze the morphology of the synthesized ZnHApD sample, a HITACHI S4500 scanning electron microscope (Hitachi, Ltd., Tokyo, Japan) equipped with an X-ray energy dispersive spectroscopy (EDX) system was used.

### 2.3. Biological Studies

#### 2.3.1. Cell Culture

An American Type Culture Collection (ATCC), HEK-293 cell line (ATCC CRL-1573), derived from human embryonic kidney cells, was grown at 37 °C in a humidified atmosphere (5% CO_2_) in a DMEM (Dulbecco’s Modified Eagle Medium) medium containing 10% (v/v) fetal bovine serum (FBS) and 1% penicillin/streptomycin. Every 2 days the culture medium was changed until cells reached confluence. After that, cells were trypsinized with 0.25% (w/v) Trypsin—0.53 mM EDTA (ethylenediaminetetraacetic acid (Sigma-Aldrich, St. Louis, MA, USA). Then, HEK-293 cells at a density of 5 × 10^5^ cells/ml were seeded in 6-well plates and incubated at different concentrations (62.5, 125, 250 and 500 μg/mL) of the ZnHApD solution for 24 and 48 h. All cell manipulation procedures were effectuated in a sterile laminar flow hood. The cell viability (%) of the treated cells was calculated in relation to the control (100%). 

#### 2.3.2. Analysis of the Actin Cytoskeleton

The HEK-293 cells were seeded on 6-well plates (5 × 10^5^ cells/well). When cells had adhered to the plates, the medium was removed and replaced by different concentrations (62.5, 125, 250 and 500 μg/mL) of the ZnHApD solution. After 24 and 48 h of treatment, the medium was removed, and the cells were fixed in 4% paraformaldehyde for 30 min at 4 °C. After fixation, the cells were washed three times with PBS (phosphate-buffered saline). Then, the cells were incubated for 30 min in PBS containing 20 μg/mL phalloidin-FITC (Phalloidin, Fluorescein Isothiocyanate Labeled) (Sigma-Aldrich, St. Louis, MO, USA). The nuclei were counterstained with 1 mg/mL DAPI (4′-6-Diamidino-2 phenylindole) solution (Invitrogen, Carlsbad, CA, USA) in 1× PBS for 5 min. Finally, the images were registered using an Olympus IX71 fluorescent microscope. 

### 2.4. Antibacterial Assays

The antibacterial activities of the ZnHApD samples were determined against ATCC reference bacterial strains that were Gram-positive (*Staphylococcus aureus* ATCC 25923, *Porphyromonas gingivalis* ATCC 33277) and Gram-negative (*Escherichia coli* ATCC 25922). The bacterial strains were cultivated in a brain heart infusion (BHI) medium enriched with hemin and vitamin K. The cultures were incubated at 37 °C under anaerobic conditions for 24 h and afterwards resuspended in the BHI medium. 

In order to evaluate the antibacterial activity of ZnHApD solutions, the bacterial solutions were cultivated in the presence of different concentrations of ZnHAp samples for 24 h and 48 h in 96-well microtiter plates. The assessment of the effect of the ZnHAp solution on the bacterial growth of *S. aureus*, *E. coli* and *P. gingivalis* bacterial strains was done by measuring the optical density at 600 nm (OD_600_). The experiments were performed in triplicate.

### 2.5. Statistical Analyses

All experiments were performed in triplicate and the measurements were repeated three times. The data was represented as mean ± standard deviation (SD). The levels were compared by the Student’s *t*-test. From a statistical point of view, *p* values of less than 0.05 were taken into account. 

## 3. Results and Discussion

The main purpose of this study was to provide information on the stability of concentrated ZnHApD dispersions. Since dense suspensions are opaque, transmission and scattering of light are subject to unwanted concentration effects such as multiple scattering. In such situations, ultrasound spectroscopy is an alternative method that can give precise information on the stability of concentrated suspensions. The most promising feature of ultrasonic measurements is their applicability to particle systems highly concentrated under unbalanced conditions.

Usually, inorganic colloidal nanoparticles are defined as very small, nanoscale objects having an inorganic core and dispersed in a solvent. Over time, various approaches have been developed for the coating of different nanoparticles with organic materials based on the surface affinity towards different chemical groups. The most often encountered mechanisms of coating nanoparticles with dextran are through noncovalent binding interactions and core-shell dissociation that may occur under certain biological conditions.

**Figure 2:**
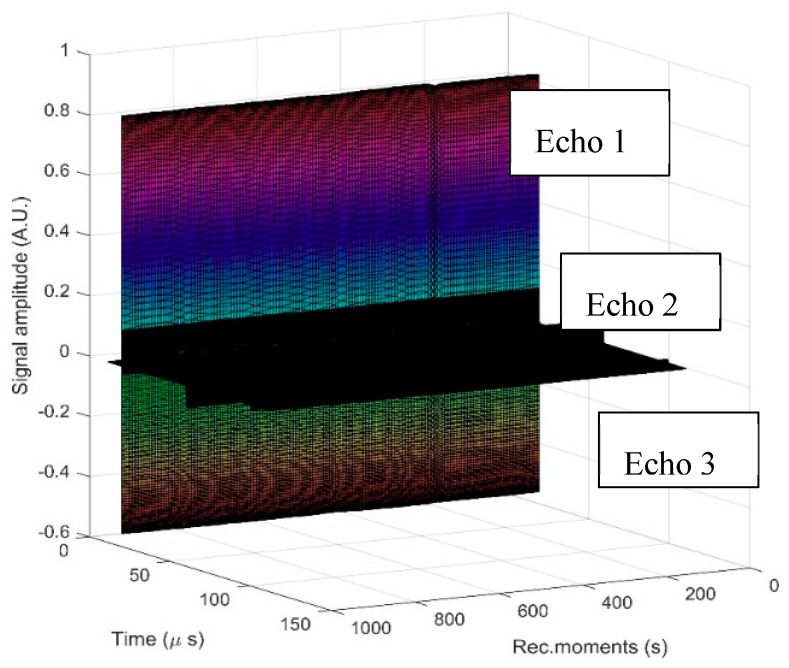
Recorded T signals during 1000 s. Each signal includes three echoes.

The analysis method proposed in this study has allowed for quantitative relative measurements to be made, thus eliminating the influence of transducer attenuation and spectrum. The recording time was 1000 s and corresponded to 200 records (Figure 2). On the other hand, each record contained 100 k samples. The three echoes obtained for the ZnHApD suspension were compared with the echoes identified in double-distilled water in the same configuration. 

The average speed in the sample was deduced by the intercorrelation of each echo with the corresponding echo in the reference fluid. Thus, for a velocity equal to c_0_ = 1489.23 m/s in the reference fluid at 22.3 °C, a mean sample (ZnHApD nanoparticles in aqueous suspension) velocity of c_s_ = 1504 m/s was deduced. The relation $c=\sqrt{\frac{1}{k\rho }}$ allows us to calculate the speed of ultrasonic waves in homogeneous liquids. The adiabatic compressibility k is the inverse of the compression elastic modulus (K = 1/k), and ρ is the averaged mass density. The averaged values for biphasic suspensions can be obtained from: $k=\Phi k_{2}+\left(1-\Phi \right)k_{1}$ and $\rho =\Phi \rho_{2}+\left(1-\Phi \right)\rho_{1}$. Φ represents the volumetric fraction of solid particles in the solvent, which is double-distilled water in this case. The pairs (k_2_, ρ_2_) of the particles in suspension can be determined if the volumetric fraction is known and the elastic properties of the solvent are also known (e.g., for water: $k=4.54\times 10^{-10\;}Pa^{-1}$). Moreover, if one of the two parameters are known, then the other one can be determined with accuracy.

**Figure 3:**
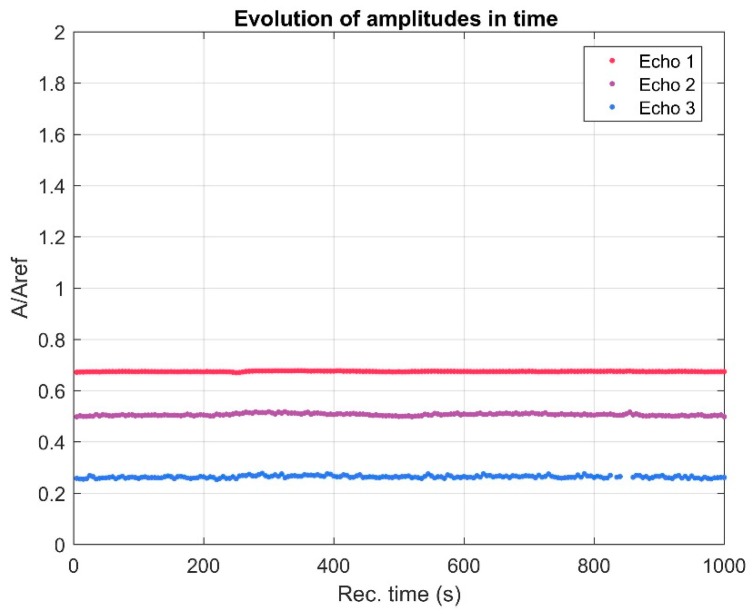
Evolution in time of the echo amplitudes.

A first information report regarding the stability of the sample is given by the evolution in time of the echo amplitudes (Figure 3). 

**Figure 4:**
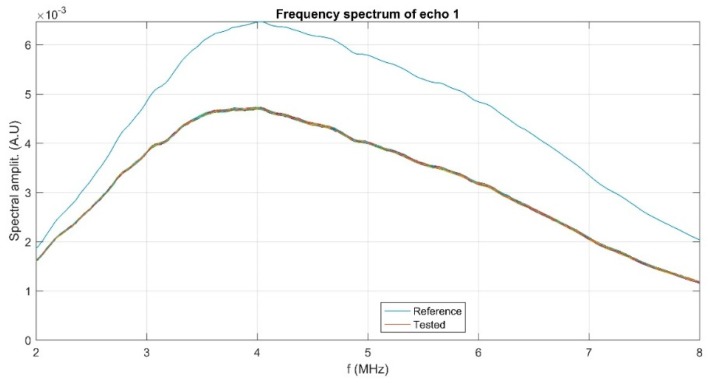
Frequency spectrum of echo 1 for all records and for the reference fluid (blue line).

As could be noticed in Figure 3, the three recorded signals indicate a stable amplitude level. In addition, the behavior of the three recorded signals is directly influenced by the physical stability of the sample. In the studies on bioceramic layers with antifungal properties [8], we proposed a stability parameter, measured in s^-1^ and defined as $s=\frac{1}{A_{m}}\left|\frac{dA}{dt}\right|$. The stability coefficient calculated for ZnHApD nanoparticles in suspension following ultrasound measurements was s = 0.00149 (1/s). The value obtained for the stability coefficient is close to that of pure water (s = 0), which shows that the studied sample has an excellent stability. From the Fourier spectrum of each echo for all records, the frequency characteristics of each echo can be determined. In Figure 4 the frequency spectrum for the first echo is presented. The peak that occurs at 4 MHz was due to transducers whose center frequency should be at 5 MHz. It can be observed that the spectrum of the 200 ultrasonic records of the first echo are almost superposed. This overlap also indicates a good stability. The decrease of the amplitudes vs. frequency compared with the reference sample was due to the attenuation in the sample.

**Figure 5:**
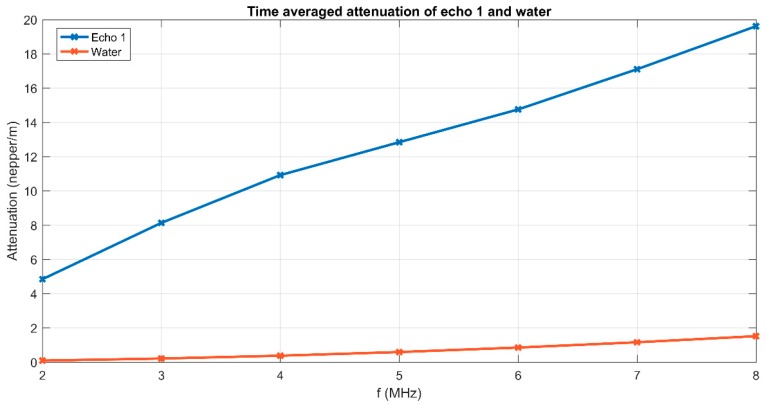
Spectrum amplitude ratios of the sample relative to the reference sample.

The calculation of the amplitude ratios between the measured sample and the reference sample (Figure 5) is useful for studying the stability of the ZnHApD suspension. On the other hand, decreasing amplitudes at higher frequencies is a phenomenon which is typical to ultrasounds waves.

**Figure 6:**
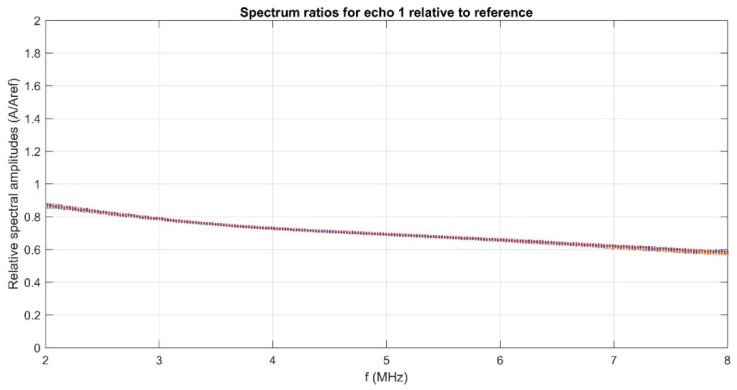
Spectral attenuation of the first echo and reference fluid.

Moreover, at each frequency in the spectrum, an average amplitude ratio was calculated relative to the reference fluid. Figure 6 shows the graphical representation of the attenuation vs. frequency for the first echo.

**Figure 7:**
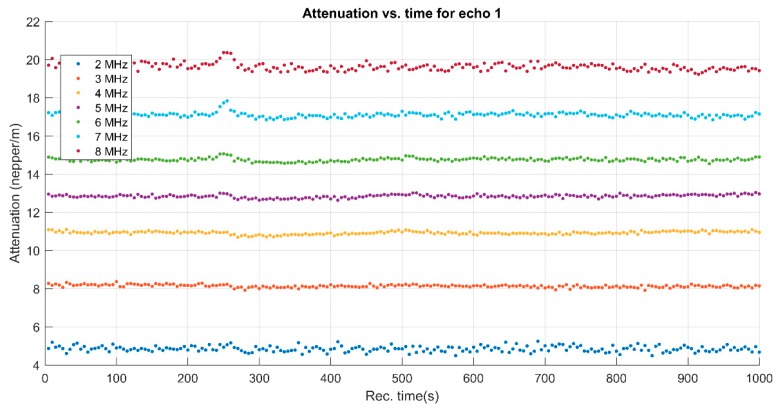
Spectral attenuation vs. time for first echo.

The attenuation was defined for two echoes of amplitudes *A*_1_ and *A*_2_, determined after traveling a distance *d*, as $\alpha =-\frac{1}{d}ln\frac{A_{2}}{A_{1}}\left[Np/m\right]$. The attenuation *α*_1_ of ultrasounds in pure water at each frequency of the spectrum was known. For the identical amplitude *A*_0_ of the wave produced by the transducer, the amplitude received by the other transducer is $A_{1}=A_{0\;}\exp\left(-\alpha_{1}d\right)$ in water and $A_{2}=A_{1\;}\exp\left(-\alpha_{2}d\right)$ in the sample at the same frequency. From the ratios of measured amplitudes, the formula $\alpha_{2}=\alpha_{1}-\frac{1}{d}ln\frac{A_{2}}{A_{1}}\left[Np/m\right]$ was followed, which was implemented in the signal analysis computer code. The attenuation of the sample was determined experimentally while the attenuation of the reference fluid was assigned from specialized literature. The attenuation in the sample was notably higher than in the reference fluid (water), varying from 4.85 Np/m at 2 MHz up to 19.62 Np/m at 8 MHz. It is very important to also note the slope that was given by the stability parameter and the possible non-linear variation of the attenuation with increasing frequency. More details could be obtained by analyzing the evolution in time of the attenuation for each of the frequencies highlighted clearly in Figure 6. The results obtained from the analysis of the evolution in time of the attenuation for each marked frequency are plotted in Figure 7. The stability of the sample is also proven by these plots, showing almost constant values of the attenuation at each frequency.

**Figure 8:**
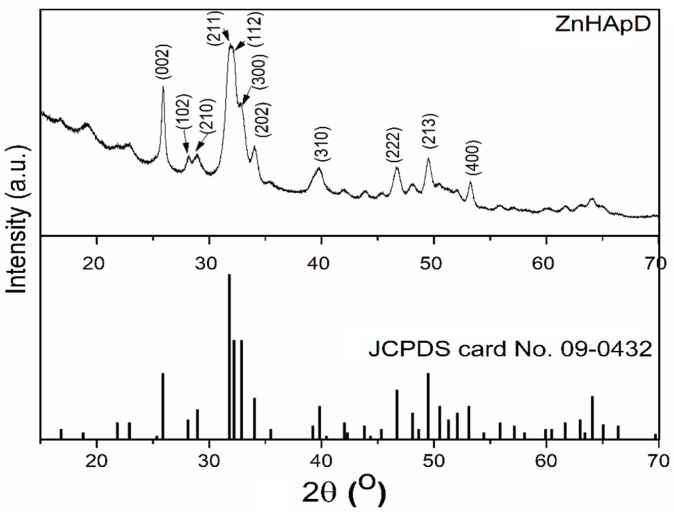
X-ray diffraction (XRD) pattern of the synthesized dextran-coated zinc-doped hydroxyapatite (ZnHApD) sample and the standard pattern of pure hexagonal HAp (JCPDS card No.09-0432). ApHA.

The crystalline structure of ZnHApD obtained by an adapted sol-gel method was evaluated by an XRD study. Figure 8 reveals the XRD pattern of the powders obtained from synthesized ZnHApD solutions and the standard pattern of pure hexagonal HAp (JCPDS (Joint Committee on Powder Diffraction Standards) card No. 09-0432). After performing the phase analysis, it was shown that the XRD maxima (Figure 8) of the synthesized sample were crystalline. Following the XRD studies, it was shown that the XRD model for the synthesized sample matched to the model of standard pure hydroxyapatite with a hexagonal crystal structure, and the space group of P6_3_/m according to JCPDS data (card No. 09-0432). The main peaks identified from the XRD pattern were indexed to (002), (102), (210), (211), (112), (300), (202), (310), (222), (213) and (400) lattice planes of the hexagonal pure HAp. The lattice parameters (a = b = 9.4307 Å and c = 6.8546 Å) of the ZnHApD sample were calculated using the XRD data. In the XRD pattern of ZnHApD, supplementary maxima were not found, which shows that no impurity that could have been generated during the cooking process was identified.

**Figure 9:**
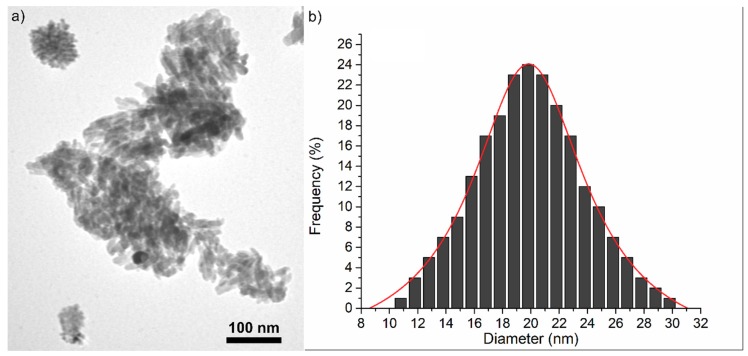
Representative image of ZnHApD powders (**a**) and size distributions (**b**) obtained by transmission electron microscopy (TEM). Scale bars = 100 nm.

In order to obtain direct information of the morphology, size and size distribution, the ZnHApD sample was investigated by TEM and SEM. TEM morphology and size distribution of ZnHApD synthesized by un adapted sol-gel method is presented in Figure 9. The particles observed in Figure 9a revealed a uniform size distribution with elongated morphology in an ellipsoidal form. The diameter varied between 9 and 31 nm (Figure 9b).

**Figure 10:**
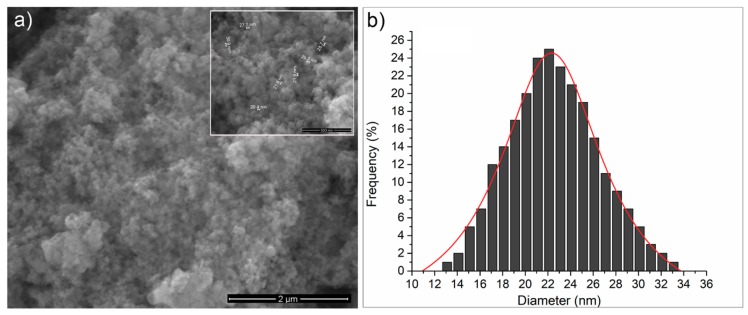
Representative image of ZnHApD powders (**a**) and size distributions (**b**) obtained by scanning electron microscopy (SEM).

**Figure 11:**
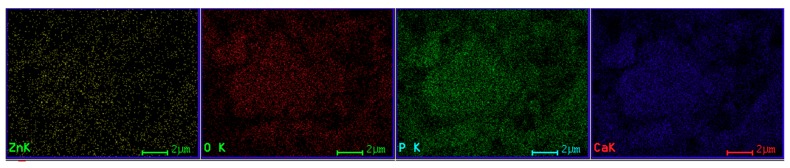
The energy dispersive spectroscopy (EDX) mappings of of ZnHApD powders.

The SEM image of the ZnHApD sample is shown in Figure 10a. The observations revealed that the particles were ellipsoidal and the particles tended to agglomerate (Figure 10a). The size distributions obtained by the SEM analysis is shown in Figure 10b. The particle size ranged from 11 to 34 nm and the average particle size was about 22 ± 0.6 nm. The EDX mappings confirmed the presence of the constituent elements (Ca, P, O, Zn) and revealed a homogeneous distribution of these in the sample (Figure 11). Both TEM and SEM investigations of the ZnHApD sample have shown that the particles have an ellipsoidal shape and have revealed a narrow distribution of sizes.

**Figure 12:**
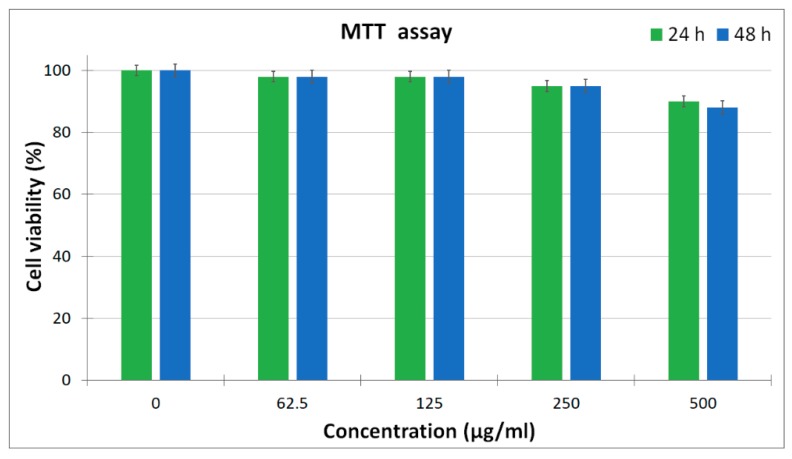
MTT (3-(4,5-Dimethylthiazol-2-yl)-2,5-Diphenyltetrazolium Bromide) assay of HEK-293 cell viability for 24 and 48 h cultured with the ZnHApD nanoparticles. All values were represented as average ± standard deviation for n = 3 per experimental condition.

Different concentrations (62.5, 125, 250 and 500 μg/mL) of the ZnHApD solution were added to HEK-293 cells and analyzed after 24 and 48 h, respectively. The results obtained are shown in Figure 12. As can be seen in Figure 12, the ZnHApD nanoparticles exhibited a non-toxic activity against HEK-293 cells at all concentrations analyzed. After 24 h, cell viability was 90–98%. At a concentration of 500 μg/mL, the cell viability was 90%, while in case of lower dosages (62.5 and 125 μg/mL) the cell viability was 98%. A 95% cell viability was observed at the concentration of 250 μg/mL. After an 48 h incubation of HEK-293 cells with different concentrations of ZnHApD solution the results concerning the cell viability were similar to those obtained at 24 h for concentrations of 62.5, 125 and 250 μg/mL. An insignificant decrease was recorded at the concentration of 500 μg/mL (88%). The results on the viability of HEK-293 cells have shown that the ZnHApD solution nanoparticles is biocompatible.

**Figure 13:**
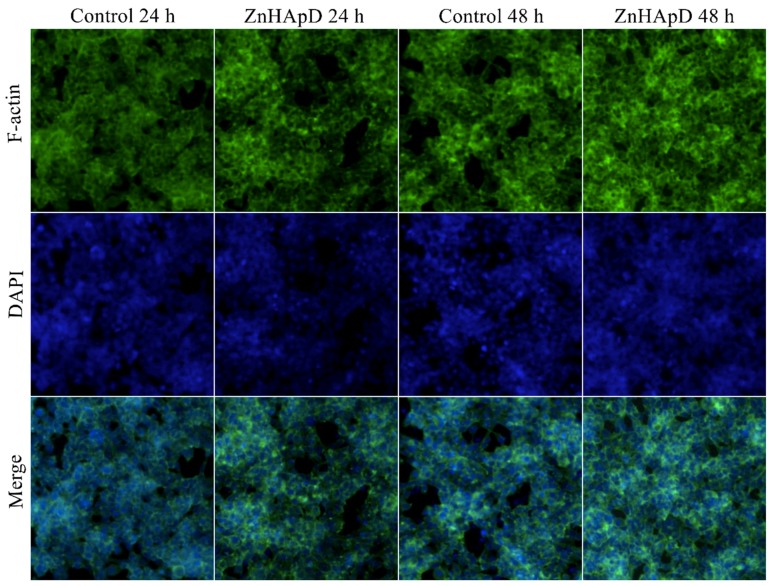
Images obtained by fluorescence microscopy after 24 and 48 h of HEK-293 cell cultured in the presence of a 500 μg/mL ZnHApD solution. Actin cytoskeleton of cells, stained with phalloidin-FITC (green). The nuclei were stained with DAPI (blue).

In order to better visualize the adhesion and proliferation of HEK-293 cells in the presence of ZnHApD, we used a staining with phalloidin-FITC for F-actin, as it is well known that the filamentous actin (F-actin) cytoskeleton is essential for maintaining the shape and structure of cells and for many physical cellular processes, including cell adhesion, migration and division [22,51,52,53,54]. A staining with DAPI was used to detect nuclei in fluorescence microscopy. Figure 13 shows the images obtained by fluorescence microscopy after 24 and 48 h of HEK-293 cell cultured in the presence of a 500 μg/mL ZnHApD solution. As can be seen in Figure 12, after 24 and 48 h of the HEK-293 cell cultured in the presence of 500 μg/mL ZnHApD solution, the cell proliferation was not affected. The actin cytoskeleton of cells shows an appropriate morphology to the control. The DAPI staining of the nuclei did not reveal differences in relation to the control at the two-time intervals at which the study was conducted.

*Staphylococcus aureus* and *Escherichia coli* are one of the most common microorganisms associated with the apparition of life treating infections [55]. *S. aureus* and *E. coli* are culpable for the majority of hospital and community-onset infections. More than that, given the appearance of antimicrobial resistant isolates, various strains are now resistant to several important antimicrobial classes [56]. On the other hand, *P. gingivalis* is one of the most aggressive bacteria found in the oral cavity. *P. gingivalis* is a pathogenic, Gram-negative, anaerobic bacterium that plays a leading role in the initiation and progression of periodontal disease, the disease leading to the loss of teeth [57]. Although not found in large amounts in the oral cavity, *P. gingivalis* leads to an uncontrolled growth of the commensal microbial community [57].

**Figure 14:**
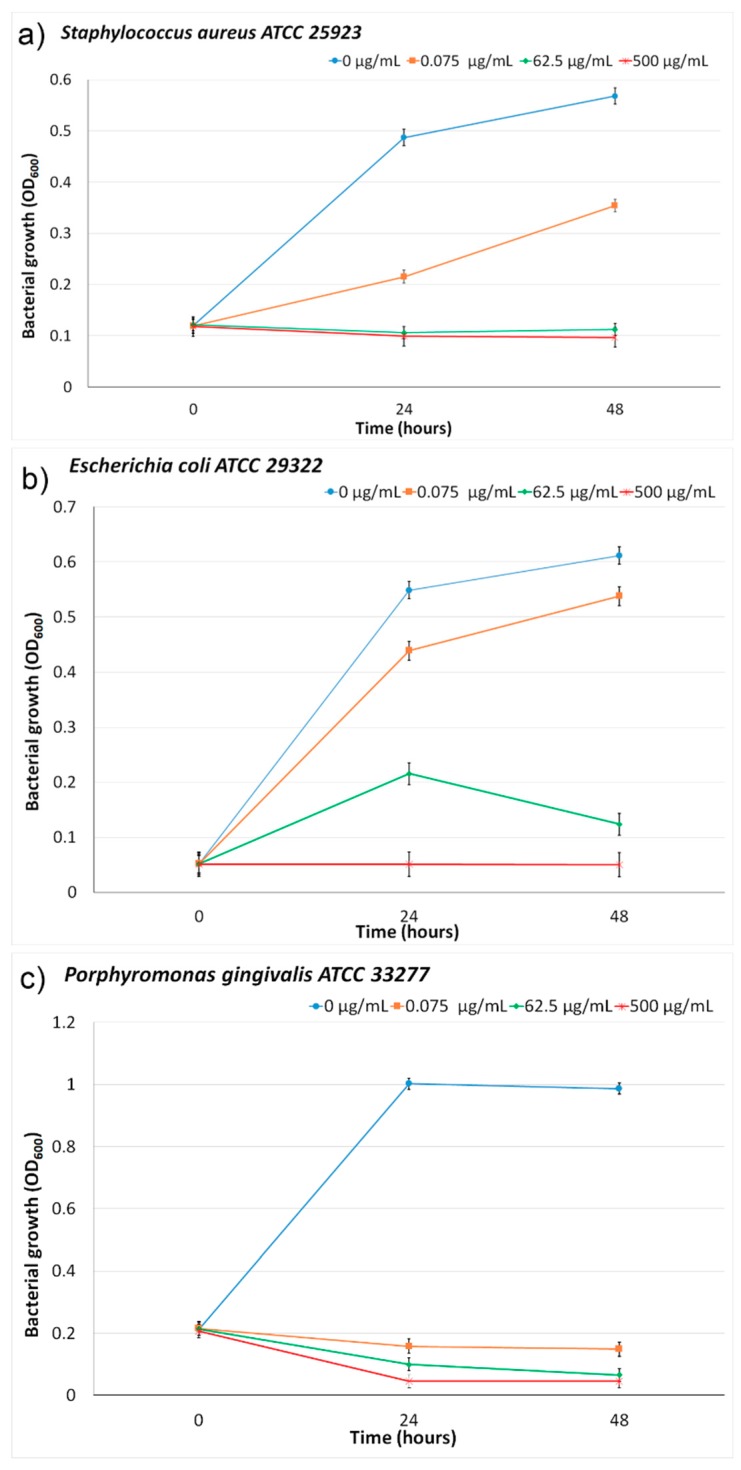
Inhibitory effects of ZnHApD solution on the growth of bacteria (mean ± SD). *Staphylococcus aureus* ATCC 25923 (**a**), *Escherichia coli* ATCC 25922 (**b**) and *Porphyromonas gingivalis* ATCC 33277 (**c**) at 24 and 48 h.

Among the tested bacteria, *P. gingivalis* ATCC 33277 was the most sensitive (Figure 14c). At 24 h and 48 h, an inhibition of growth of *P. gingivalis* ATCC 33277 in the presence of 0.075 μg/mL ZnHApD was observed. For *P. gingivalis* ATCC 33277 a medium with 500 μg/mL ZnHApD suppressed bacterial growth in the first 24 h. In the presence of a medium with 62.5 μg/mL ZnHApD, the bacterial growth was suppressed after 48 h. For *S. aureus* ATCC 25923 (Figure 14a), a decrease in bacterial growth was observed in the first 24 h in the presence of a medium of 62.5 μg/mL and 500 μg/mL, but the inhibitory effect of ZnHApD was not complete at any time (24 or 48 h). For *E. coli* ATCC 25922 (Figure 14b) an inhibition of bacterial growth in the first 24 h was observed in the presence of a medium of 500 μg/mL of ZnHApD. At 48 h, a decrease in bacterial growth was observed both in the presence of a medium with 62.5 μg/mL and 500 μg/mL of ZnHApD. The inhibitory effect of 62.5 μg/mL and 500 μg/mL ZnHApD on the *E. coli* ATCC 25922 bacterial strain was not complete.

It is well known that the treatments that have been realized so far for the prevention of infection with these bacteria (*S. aureus*, *E. coli* and *P. gingivalis*) or to treat infections produced by these bacteria with systemic or local antibiotics are only partially effective. Moreover, studies conducted a few years prior to the current study [58,59,60,61] have shown that these bacteria can become resistant to antibiotics when administered over a long period of time. Therefore, finding new solutions that lead to good efficiency in preventing and treating these infections is a great challenge. The development of zinc-doped nano HAp in a dextran matrix could play an important role in biomedical domains as HAp plays an important role in the process of differentiation and mineralization of bone cells, while zinc can minimize bacterial adherence. Furthermore, Gibbons et al. [61] have shown that dextran is relatively resistant to the attack of salivary microorganisms. Since in orthopedics and dentistry postoperative infections are a major problem, this material could play an important role in reducing these issues. In this context, studies on the efficacy of ZnHApD solutions on reducing the microbial activity of three of the most involved bacteria in postoperative orthopedic and dental infections (*S. Aureus*, *E. coli* and *P. gingivalis*) could bring about new information on these materials that could be used to prevent these infections in future.

## 4. Conclusions

The concentrated ZnHApD solution was obtained by an adapted sol-gel method. The stability of the sample was proven by analyzing the evolution in time of the attenuation for each frequency, the attenuation values being constant at each frequency. The stability coefficient calculated for the concentrated ZnHApD solution ion was close to that of pure water. The XRD pattern exhibited peaks belonging to the structure of hydroxyapatite and no impurities was found. The TEM and SEM examinations of the ZnHApD sample showed that the particles have an ellipsoidal shape and a narrow distribution of sizes. The non-toxic activity of the ZnHApD nanoparticles on HEK-293 cells was revealed at all analyzed concentrations. At the two-time intervals at which the present study was conducted, HEK-293 cell morphology remained unchanged from the control. The results of the antibacterial assay revealed that all the tested microorganisms were inhibited by the ZnHApD solution after 24 and 48 h of incubation. Nevertheless, bacterial adhesion is usually strongly affected under dynamic conditions and most often the results of an in vitro study needs to be validated under dynamic conditions or with the aid of in vivo experiments. The action of ZnHApD on bacteria remains a focal area for ulterior investigations. The study of the effects of zinc ions could be used both to make the best use of their antibacterial activity and to reduce the concentrations necessary to obtain a certain antibacterial effect for food safety and health purposes. The results presented in this study open new opportunities regarding the development of alternative preventive strategies to combat implant-related microbial infection.
